# Triple-negative, basal-like, and quintuple-negative breast cancers: better prediction model for survival

**DOI:** 10.1186/1471-2407-10-507

**Published:** 2010-09-23

**Authors:** Yoon-La Choi, Ensel Oh, Sarah Park, Yeonju Kim, Yeon-Hee Park, Kyoung Song, Eun Yoon Cho, Yun-Chul Hong, Jong Sun Choi, Jeong Eon Lee, Jung Han Kim, Seok Jin Nam, Young-Hyuck Im, Jung-Hyun Yang, Young Kee Shin

**Affiliations:** 1 Laboratory of Cancer Genomics and Molecular Pathology, Samsung Medical Center, Seoul, Korea; 2 Department of Pathology, Samsung Medical Center, Sungkyunkwan University School of Medicine, Seoul, Korea; 3 Research Institute of Pharmaceutical Science, Department of Pharmacy, Seoul National University, Seoul, Korea; 4 Interdiciplinary Program of Bioinformatics, Department of Natural Science, Seoul National University, Seoul, Korea; 5 Division of Medical Oncology, Department of Internal Medicine, Seoul St. Mary's hospital, Catholic University, Seoul, Korea; 6 Cancer Early Detection Branch, National Cancer Control Research Institute, National Cancer Center, Goyang, Korea; 7 Department of Internal Medicine, Samsung Medical Center, Sungkyunkwan University School of Medicine, Seoul, Korea; 8 Department of Preventive Medicine, Seoul National University College of Medicine, Seoul, Korea; 9 Department of Pathology, Dongguk University-Seoul, Graduate School of Medicine, Seoul, Korea; 10 Division of Breast and Endocrine Surgery, Department of Surgery, Samsung Medical Center, Sungkyunkwan University School of Medicine, Seoul, Korea

## Abstract

**Background:**

Triple-negative breast cancers (TNBCs) and basal-like breast cancers (BLBCs) are known as poor outcome subtypes with a lack of targeted therapy. Previous studies have shown conflicting results regarding the difference of prognostic significance between TNBCs and BLBCs. In this study, we aimed to characterize the prognostic features of TNBCs, in view of BLBCs and quintuple-negative breast cancers (QNBC/5NPs).

**Methods:**

Using tissue microarray-based immunohistochemical analysis, we categorized 951 primary breast cancers into four or five subtypes according to the expression of ER, PR, HER2, and basal markers (CK5/6, EGFR).

**Results:**

The results of this study showed that both TNBCs and BLBCs were associated with high histological and/or nuclear grades. When the TNBCs are divided into two subtypes by the presence of basal markers, the clinicopathologic characteristics of TNBCs were mainly maintained in the BLBCs. The 5-subgrouping was the better prediction model for both disease free and overall survival in breast cancers than the 4-subgrouping. After multivariate analysis of TNBCs, the BLBCs did not have a worse prognosis than the QNBC/5NPs. Interestingly, the patients with BLBCs showed significant adjuvant chemotherapy benefit. In addition, QNBC/5NPs comprised about 6~8% of breast cancers in publicly available breast cancer datasets

**Conclusion:**

The QNBC/5NP subtype is a worse prognostic subgroup of TNBCs, especially in higher stage and this result may be related to adjuvant chemotherapy benefit of BLBCs, calling for caution in the identification of subgroups of patients for therapeutic classification.

## Background

Breast cancer is a heterogeneous disease, and basal-like breast cancers (BLBCs) have attracted particular attention because they have been shown to have shorter survival compared to the other subtypes [1,2]. With the practical difficulties of gene-expression profiling as a routine diagnostic tool, immunohistochemical surrogate panels have been introduced to identify BLBCs that are 'triple-negative breast cancers' (TNBCs) (estrogen receptor (ER)-negative, progesterone receptor (PR)-negative, HER2 not overexpressed) [2,3]. The immunohistochemical panel that is considered to be the gold standard in the identification of BLBCs was proposed by Nielsen et al., in which BLBCs are defined as breast cancers expressing neither ER/PR nor HER2 and expressing CK5/6 and/or EGFR [2]. Both TNBCs and BLBCs have attracted clinical interest in regard to their prognoses and associated therapeutic approaches [4]. Several clinical trials have been conducted on the routine diagnostic immunohistochemical assay of TNBCs [5]. However, it should be noted that identification of a subgroup of tumors based solely on the lack of expression of immunohistochemical markers affords the risk of incorrect assignment due to technical artifacts [6].

Recently, Kreike et al. examined the gene expression and pathological characteristics of 97 TNBCs and concluded that "BL tumors can be reliably defined by TN immunohistochemistry (IHC)" and that "TN tumors are synonymous with BL tumors" [7]. However, Rakha et al. mentioned that equating TNBC with BLBC is misleading, and insisted that while most TNBCs fall into the BL molecular subtype of breast cancer, the terms are not completely synonymous [8]. Many studies have shown that TNBCs have the worst prognosis without the option of chemotherapy. On the other hand, other studies have shown that the expression of "basal markers" (CK5/6, CK14, CK17, and/or EGFR) is associated with a poor prognosis [9-11]. Significantly, Rakha et al. and Cheang et al. reported that BLBCs defined by five biomarkers showed worse response to chemotherapy, and shorter survival [12,13].

Therefore, the aim of our study was to compare prognosis between models in which breast cancers were divided by four subtypes (including TNBC) and five subtypes (including BLBC), and to define the associated clinicopathological factors. To perform this study, we designed a longitudinal study, and used immunohistochemical markers to identify breast tumor intrinsic subtypes using formalin-fixed, paraffin-embedded (FFPE) tumor blocks, and examined the clinicopathologic parameters of BLBCs and TNBCs. Also, we explored BLBC and TNBC in two large microarray datasets by comparing the expression levels of the five markers according to the subtypes.

## Methods

### Study population

We collected breast cancer cases from the Samsung Medical Center in Seoul, Korea. Inclusion criteria for this study were: 1) histologically verified incident breast cancer; 2) female; 3) between 20-80 years of age; 4) enrolled between January 1, 1995 and December 31, 2002; 5) breast tissue samples available for study. All of the subjects were diagnosed stage I to III primary breast cancer and underwent surgical treatment and adjuvant chemotherapy according to standard treatment protocols. Radiation therapy was performed in the cases of breast conserving surgery or on patients with stage III breast cancer. All ER-positive patients underwent hormonal therapy with tamoxifen. No patients underwent anti-HER-2 therapy. We reviewed each patient's medical record for clinical information, including follow-up status and outcome information. Breast cancer stage was classified according to the American Joint Committee on Cancer (AJCC) TNM criteria (6th edition). In the case of live subjects, the last date of follow-up was June 30, 2007. The protocol for the study was approved by the institutional review board (IRB) of the Samsung Medical Center.

### Immunohistochemistry and fluorescent in situ hybridization

The original H&E-stained slides from the patients in the retrospective cohort were reviewed, and representative tumor regions without secondary change, such as hemorrhage, necrosis, and fibrosis, were marked by a pathologist (YL Choi). Corresponding 1290 FFPE breast cancer tissue blocks were obtained. Two 2 mm cores from each case were obtained, and two sets of tissue microarray (TMA) paraffin blocks were made. The sections were deparaffinized with xylene, hydrated in serial dilutions of alcohol, and then immersed in 3% hydrogen peroxide solution to neutralize endogenous peroxidase activity. Next, sections were microwaved in citrate buffer for antigen retrieval. Slides were incubated with monoclonal antibodies against CK5/6 (1:100, M7237, DAKO, Carpinteria, CA, USA), HER2 (1:250, A0485, DAKO), and EGFR (1:30, M7239, Novocastra) for 1 hour at room temperature. After washing, the tissue section was reacted with the biotinylated anti-mouse secondary antibody, followed by incubation with streptavidin-horseradish-peroxidase complex. Slides were washed, and the chromogen was developed for 5 minutes with liquid 3,3'-diaminbenzidine (DAKO). HER2 fluorescent in situ hybridization assay was performed with the PathVysion HER2 DNA Probe Kit (Abbott Molecular, Inc.) according to the manufacturer's instructions. The average copy number for each probe was determined and the amplification ratio was calculated as a ratio between the average copy per cell for HER2 and the average copy number for centromere 17.

Two pathologists (YL Choi, JS Choi) were blinded to the clinical outcomes of the patients, and independently scored the results of the staining. ER and PR stain data were acquired from the pathologic report. The staining studies were scored using the Allred score (AS), a method that semi-quantitates the proportion of positive cells (scored on a 0 to 5 scale) and staining intensity (scored on a 0 to 3 scale), with a maximum score of 8; an AS > 2 was considered positive [14]. The CK5/6, EGFR and HER2 immunohistochemical results were from TMA and considered positive with the following criteria in at least one core. CK5/6 stains were considered positive if any cytoplasmic and/or membranal staining was observed. Immunostaining for EGFR was interpreted as positive when at least 10% of the tumor cells showed moderate to strong membranal staining [15]. HER2 positivity was defined as an intensity of 3+ by IHC or as gene amplification ratio of ≥ 2.0 by FISH in the case of an intensity of 1+ or 2+ by IHC [16].

### Definition of breast cancer subtypes by immunohistochemistry

The immunohistochemical surrogate panel (ER, PR, HER2, EGFR, and CK5/6) used to define the breast cancer subtypes has been previously published [2,17]. In this study, we used two subtyping schemes. Each case was classified as one of five IHC-based subtypes: luminal A (ER+ and/or PR+, HER2−), luminal B (ER+ and/or PR+, HER2+), HER2 (ER−, PR−, and HER2+), and TNBC (ER-, PR-, and HER2-) [17]. TNBCs were further divided into BLBCs and QNBC/5NPs according to the basal-markers. TNBC expressing either EGFR or CK5/6 was defined as BLBC (ER−, PR−, HER2−, CK5/6+, and/or EGFR+). Breast tumors which were TN and expressed neither CK5/6 nor EGFR were defined as 'quintuple-negative breast cancer' (QNBC/5NP) (ER−, PR−, HER2−, CK5/6−, and EGFR−).

### Statistical Analysis

Disease free survival (DFS) was defined as the time from the date of diagnosis to the date of the documentation of relapse, including locoregional recurrence and/or distant metastasis. Overall survival (OS) was expressed as the number of months from diagnosis to the date of death. Differences in the frequencies of basic characteristics, clinical parameters, and subtypes were statistically analyzed using the chi-square test, or Fisher's exact test in the case of less than five expected cases. For multiple statistical comparisons, chi-square test was corrected by Bonferroni's correction. Survival curves were constructed using the Kaplan-Meier method, and the log-rank test was used to compare mean survival rates across subtypes. For multivariate analysis, Cox regression models were built to estimate the adjusted hazard ratios (HRs) of breast cancer subtypes with tumor size, lymph node involvement and adjuvant chemotherapy. To test the statistical significance between model 1 (5-subgrouping) and model 2 (4-subgrouping), a likelihood ratio test of the differences was used. The null hypothesis was that the model 2 did not predict survival differently than model 1. Statistical significance was defined as P < 0.05. All statistical analyses were performed using SPSS 15.0 and SAS 9.1 statistical software packages.

### Microarray Analysis

We classified TNBC into BLBC and QNBC/5NP with two public independent gene expression datasets, Vijver et al.(316 samples) and Wang et al.(286 samples) [18-20] Vijver et al. was generated with 2-color oligo chips (Agilent, Hu25K) and Wang et al. was 1-color oligo chips (Affymetrix, U133X3P). Each dataset consists of a large number of random breast cancer patients. Vijver et al. is available at http://www.rii.com/publications/2002/nejm.html, and Wang et al. can be downloaded from the NCBI GEO data repository (GSE2034). For Wang et al, gene expression values were centered by subtracting the mean value of each probe set across the samples from each measured value. Both datasets included only ER IHC information, so the other four IHC results (PR, HER2, CK5/6 and EGFR) were dichotomized into 'positive (+)' and 'negative (-)' by the mRNA expression levels of the corresponding genes on the microarray chips. Under the assumption that Cheang et al.'s cohort with 4,046 breast samples was representative of a random sample of breast cancer population and the proportion of ER IHC result (ER+: 70.5%) was similar to the ER IHC results (Vijver et al.: 76%, Wang et al.: 72%) in the selected microarray data sets, we used the proportion of the status of the IHC results of each marker in his dataset to determine the cut-off for the surrogate mRNA expression for the selected microarray datasets [13]. For instance, the cut-off for KRT5 which corresponds to CK5/6 was determined at the point where the proportion of '+' to '-' was the same as the proportion of 'CK5/6 +' to 'CK5/6-' in Cheang et al.'s IHC results (Additional File 1, Figure S1). The cut-offs for PGR, KRT5 and EGFR were determined by synchronizing the proportion of their statuses in Cheang et al.'s IHC results and in each microarray dataset (Additional File 1, Figure S1). The cut-off for ERBB2 was determined from the clear bimodal distribution, by assigning '+' for right side and '-' for left side (Additional File 1, Figure S1). The only available IHC result, ER, was not replaced by the expression of ESR1. Each sample from the microarray datasets was assigned to one of the five subtypes according to the status of the five markers.

## Results

### Patients Characteristics

After excluding 339 (26.3% (339/1290)) cases due to failure of staining, 951 cases that had informative immunohistochemical results were included in the study. The median age in the study population at diagnosis was 47 years (range, 20-80 years). The clinicopathologic characteristics of patients are summarized in Table 1. Ductal histology was the most prevalent breast cancer type, and was present in 91.6% (872/951). Lobular histology was present in 2.7% (26/951). The remaining 5.7% (53/951) had cancers of other histological types, including mucinous, tubular, medullary, and metaplastic types. Mastectomy was performed in 62.3% (592/951), and 37.7% (359/951) underwent breast conserving surgery. Out of the 951 patients, 83.7% (796/951) received adjuvant chemotherapy; 491 were treated with nonanthracycline-based chemotherapy - CMF (cyclophosphamide, methotrexate, 5-fluorouracil) and 194 were treated with anthracycline-based chemotherapy - AC (doxorubicin, cyclophosphamide) and FAC (5-fluorouracil, doxorubicin, cyclophosphamide). Remaining 155 patients (16.3% (155/951)) did not receive any adjuvant systemic chemotherapy. The median follow-up time was 75.0 months (from 2.47 to 152.1 months).

**Table 1:** Clinicopathologic characteristics of breast cancer subtypes

			Molecular Subtyping													
					
	All								Five subtypes				Four subtypes		P value between five subtypes *,†	P value between four subtypes **,†
												
Variables			IHC-Luminal A		IHC-Luminal B		IHC-HER2		IHC-BLBC		IHC-QNBC/5NP		IHC-TNBC			
						
	N = 951		N = 486 51.1%		N = 123 12.9%		N = 113 11.9%		N = 139 14.6%		N = 90 9.5%		N = 229 24.1%			
						
	No.	(%)	No.	(%)	No.	(%)	No.	(%)	No.	(%)	No.	(%)	No.	(%)		
Age Group (years)															< 0.001	< 0.001
> 50	590	62.0	297	61.1	87	70.7	51	45.1	97	69.8	58	64.4	155	67.7		
≥ 50	361	38.0	189	38.9	36	29.3	62	54.9	42	30.2	32	35.6	74	32.3		
Family history of breast cancer															0.006	0.086
No	916	96.3	470	96.7	121	98.4	111	98.2	126	90.6	88	97.8	214	93.4		
Yes	35	3.7	16	3.3	2	1.6	2	1.8	13	9.4	2	2.2	15	6.6		
Tumor Size															0.014	0.006
≤ 2 cm	392	41.2	228	46.9	39	31.7	35	31.0	52	37.4	38	42.2	90	39.3		
2-5 cm	493	51.8	231	47.5	75	61.0	64	56.6	76	54.7	47	52.2	123	53.7		
> 5 cm	66	6.9	27	5.6	9	7.3	14	12.4	11	7.9	5	5.6	16	7.0		
N Staging															0.178	0.014
N0	498	52.4	246	50.6	52	42.3	63	55.8	89	64.0	48	53.3	137	59.8		
N1	247	25.9	131	27.0	40	32.5	22	19.5	31	22.3	23	25.6	54	23.6		
N2	118	12.4	64	13.2	19	15.4	13	11.5	12	8.6	10	11.1	22	9.6		
N3	88	9.2	45	9.3	12	9.8	15	13.3	7	5.0	9	10.0	16	7.0		
AJCC stage															0.014	0.008
I	254	26.7	148	30.5	19	15.4	22	19.5	38	27.3	27	30.0	65	28.4		
II	467	49.1	221	45.5	69	56.1	57	5.04	78	56.1	42	46.7	120	52.4		
III	230	24.2	117	24.1	35	28.5	34	30.1	23	16.5	21	23.3	44	19.2		
LN involvement															0.016	0.020
Negative	498	52.4	246	50.6	52	42.3	63	55.8	89	64.0	48	53.3	137	59.8		
Positive	453	47.6	240	49.4	71	57.7	50	44.2	50	36.0	42	46.7	92	40.2		
Nuclear Grade															< 0.001	< 0.001
Low	92	9.7	67	13.8	5	4.1	3	2.7	9	6.5	8	8.8	17	7.4		
Intermediate	486	51.1	308	63.4	65	52.8	40	35.4	26	18.7	47	52.2	73	31.9		
High	373	39.2	111	22.8	53	43.1	70	61.9	104	74.8	35	38.9	139	60.7		
Histological Grade															< 0.001	< 0.001
Well	100	10.5	63	13.0	10	8.1	11	9.7	8	5.8	8	8.9	16	7.0		
Moderate	597	62.8	334	68.7	76	61.8	65	57.5	71	51.1	51	56.7	122	53.3		
Poor	254	26.7	89	18.3	37	30.1	37	32.7	60	43.2	31	34.4	91	39.7		
Estrogen Receptor (ER)															< 0.001	< 0.001
Negative	364	38.3	11	2.3	11	8.9	113	100.0	139	100.0	90	100.0	229	100.0		
Positive	587	61.7	475	97.7	112	91.1	0	0.0	0	0.0	0	0.0	0	0.0		
Progesterone Receptor (PR)															< 0.001	< 0.001
Negative	522	54.9	143	29.4	37	30.1	113	100.0	139	100.0	90	100.0	229	100.0		
Positive	429	45.1	343	70.6	86	69.9	0	0.0	0	0.0	0	0.0	0	0.0		
HER2																
Negative	715	75.2	486	100.0	0	0.0	0	0.0	139	100.0	90	100.0	229	100.0		
Positive	236	24.8	0	0.0	123	100.0	113	100.0	0	0.0	0	0.0	0	0.0		
CK 5/6																
Negative	820	86.2	474	97.7	123	100.0	103	91.2	30	21.6	90	100.0	120	52.4		
Positive	131	13.8	12	2.5	0	0.0	10	8.8	109	78.4	0	0.0	109	47.6		
EGFR																
Negative	821	86.3	471	96.9	119	96.7	92	81.4	49	35.3	90	100.0	139	60.7		
Positive	130	13.7	15	3.1	4	3.3	21	18.6	90	64.7	0	0.0	90	39.3		
Histological type																
Invasive ductal carcinoma	872	91.6	438	90.1	122	99.2	106	93.8	125	89.9	81	90.0	206	90.0		
Invasive lobular carcinoma	26	2.7	22	4.5							4	4.4	4	1.7		
Mucinous carcinoma	13	1.4	10	2.1			2	1.8			1	1.1	1	0.4		
Invasive papillary carcinoma	8	0.8	5	1.0	1	0.8			2	1.4			2	0.9		
Medullary carcinoma	10	1.1	1	0.2			1	0.9	8	5.8			8	3.5		
Metaplastic carcinoma	6	0.6	1	0.2					3	2.2	2	2.2	5	2.2		
Others	18	1.6	9	1.9			3	2.7								
Operation																
BCS	359	37.7	200	41.2	40	32.5	30	26.5	58	41.7	31	34.4	89	38.9	0.048	0.038
Mastectomy	592	62.3	286	58.8	83	67.5	83	73.5	81	58.3	59	65.6	140	61.1		
Adjuvant chemotherapy																
Not done	155	16.3	95	19.5	20	16.3	22	19.5	10	7.2	8	8.9	18	7.9	0.004	0.002
Done	796	83.7	391	80.5	103	83.7	91	80.5	129	92.8	82	91.1	211	92.1		
Anthracycline	292	36.7	156	39.9	40	38.8	33	36.3	34	11.6	29	35.4	63	29.9	0.101	0.114
CMF	491	61.7	229	58.6	61	59.2	58	63.7	92	71.3	51	62.2	143	67.8		
Others	13	1.6	6	1.5	2	1.9	0	0	3	2.3	2	2.4	5	2.4		
Radiation therapy																
Not done	503	52.9	250	51.4	73	59.3	63	55.8	69	49.6	48	53.3	117	51.1	1.0	1.0
Done	448	47.1	236	48.6	50	40.7	50	44.2	70	50.4	42	46.7	112	48.9		

* Five subtypes are Luminal A, Luminal B, HER2+, BLBC, and QNBC

** Four subtypes are Luminal A, Luminal B, HER2+-, and TNBC

† Chi-square test using Bonferroni's correction

IHC: immunohistochemistry

### Clinicopathologic characteristics of breast cancer subtypes

51.1% (486/951) were luminal A subtype, 12.9% (123/951) were luminal B subtype, 11.9% (113/951)) were HER2 subtype, and 24.1% (229/951) were TN-subtype (Table 1). Among the TN-subtype, 139 were defined as BLBCs (14.6% (139/951)), whereas 90 cases were defined as QNBC/5NPs 90 (9.5% (90/951)). The clinicopathologic characteristics of each breast cancer subtype are shown in Table 1. Women with luminal A subtypes showed lower nuclear and histologic grade. Women with tumors of the TNBCs were younger, and less often had lymph node involvement, but had higher nuclear and poorer histological grades. When the TNBCs were subdivided into BLBCs and QNBC/5NPs according to the basal-markers, women with BLBCs maintained the features of TNBCs, showing younger age, less often lymph node involvement, higher nuclear and poorer histological grades. 8 cases out of 10 medullary carcinomas were assigned to BLBCs and 5 out of 6 metaplastic carcinomas were to TNBCs (3 in BLBCs and 2 in QNBC/5NPs). Histologic features of representative case of QNBC/5NPs and BLBCs are described (Additional File 1, Figure S2).

### Disease free survival and overall survival by breast cancer subtypes

**Figure 1:**
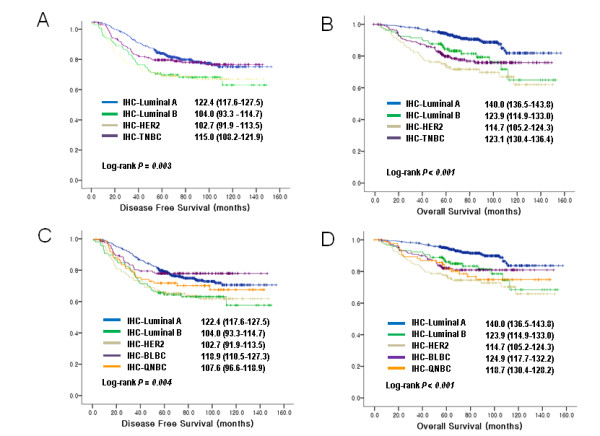
**Kaplan-Meier curves of disease-free survival and overall survival**. Disease-free survival according to (A) four and (C) five IHC-based subtypes of breast cancers in the study subjects. Overall survival according to (B) four and (D) five subtypes of breast cancers in the study subjects (Estimated mean survival with 95% CI).

DFS time ranged from 0 to 152.1 months with median of 67.5 months. During the study period, 256 women (26.9% (256/951)) had local recurrence and/or metastasis. HR and 95% confidence intervals (95% CI) for DFS according to breast cancer subtypes are shown in Table 2 and survival analyses were demonstrated in Figure 1A and 1C. Advanced stage did not significantly increase the recurrence risk of luminal B and HER2 subtype (Table 2, Additional File 1, Figure S3). Interestingly, BLBCs showed obvious DFS benefit with adjuvant chemotherapy (HR, 0.24; 95% CI, 0.10-0.60; P = 0.002), whereas TNBCs did not show statistical benefit from chemotherapy.

**Table 2:** Hazard ratios of breast cancer disease-free survival for several basic characteristics by breast cancer subtyping

			Molecular subtyping											
			
	All								Five subtypes				Four subtypes	
										
Variables	(N = 951)		IHC-Luminal A (N = 486)		IHC-Luminal B (N = 123)		IHC-HER2 (N = 113)		IHC-BLBC (N = 139)		IHC-QNBC/5NP (N = 90)		IHC-TNBC (N = 229)	
		
	HR(N of event)		HR(N of event)		HR(N of event)		HR(N of event)		HR(N of event)		HR(N of event)		HR(N of event)	
	(95%CI)	p-value	(95%CI)	p-value	(95%CI)	p-value	(95%CI)	p-value	(95%CI)	p-value	(95%CI)	p-value	(95%CI)	p-value
Age Group (yr)														
> 50	1.00(174/590)		1.00(79/297)		1.00(32/87)		1.00(25/51)		1.00(22/97)		1.00(16/58)		1.00(38/155)	
≥ 50	0.73(82/361)		0.72(38/189)		0.88(12/36)		0.42(16/62)		0.70(7/42)		1.07(10/32)		0.89(17/74)	
	(0.55-0.96)	0.020	(0.49-1.07)	0.856	(0.45-1.71)	1.0	(0.21-0.79)	0.056	(0.29-1.60)	1.0	(0.48-2.37)	1.0	(0.50-1.58)	1.0
Tumor size (cm)														
≤ 2 cm	1.00(77/392)		1.00(38/228)		1.00(16/39)		1.00(8/35)		1.00(9/52)		1.00(6/38)		1.00(15/90)	
2-5 cm	1.62(146/493)		1.86(23/75)		0.73(23/75)		1.93(25/64)		1.04(14/76)		2.72(17/47)		1.59(31/123)	
	(1.23-2.14)	0.001	(1.25-2.77)	0.016	(0.38-1.38)	1.0	(0.87-4.27)	0.864	(0.45-2.39)	1.0	(1.07-6.89)	0.288	(0.86-2.94)	1.0
> 5cm	3.14(33/66)		3.32(12/27)		1.34(5/9)		2.65(7/14)		4.04(6/11)		4.67(3/5)		4.30(8/16)	
	(2.08-4.72)	< 0.001	(.1.73-6.35)	< 0.001	(0.49-3.67)	1.0	(0.96-7.30)	0.480	(1.43-11.22)	0.064	(1.16-18.22)	0.240	(1.90-9.82)	0.008
LN involvement														
Negative	1.00(85/503)		1.00(31/248)		1.00(15/53)		1.00(18/64)		1.00(12/89)		1.00(9/49)		1.00(21/138)	
Positive	2.54(171/448)		3.28(86/238)		1.60(29/70)		1.88(22/49)		2.75(17/50)		2.83(17/41)		2.83(34/91)	
	(1.96-3.30)	< 0.001	(2.17-4.95)	0.001	(0.86-2.98)	0.980	(1.01-3.51)	0.376	(1.32-5.77)	0.045	(1.26-6.36)	0.096	(1.64-4.89)	< 0.001
AJCC stage														
I	1.00(36/254)		1.00(14/148)		1.00(8/19)		1.00(4/22)		1.00(5/38)		1.00(5/27)		1.00(10/65)	
II	1.62(106/467)		2.62(50/221)		0.55(18/69)		2.02(19/57)		1.24(13/78)		0.79(6/42)		1.03(19/120)	
	(1.02-2.14)	0.001	(1.45-4.74)	0.008	(0.24-1.26)	1.0	(0.68-5.94)	1.0	(0.44-3.48)	1.0	(0.24-3.48)	1.0	(0.48-2.20)	1.0
III	3.14(114/230)		5.86(53/117)		1.32(18/35)		3.59(17/34)		4.31(11/23)		6.30(15/21)		5.14(26/44)	
	(2.08-4.72)	< 0.001	(3.24-10.55)	0.001	(0.58-3.04)	1.0	(1.21-10.67)	0.176	(1.49-12.41)	0.056	(2.27-17.5)	0.001	(2.47-10.66)	< 0.001
Adjuvant chemotherapy														
Not done	1.00(43/155)		1.00(17/95)		1.00(9/20)		1.00(10/22)		1.00(6/10)		1.00(1/8)		1.00(7/18)	
Done	0.98(213/796)		1.57(100/391)		0.63(35/103)		0.69(30/91)		0.24(23/129)		3.13(25/82)		0.59(48/211)	
	(0.71-1.36)	0.915	(0.94-2.64)	0.083	(0.31-1.32)	0.226	(0.34-1.42)	0.312	(0.10-0.60)	0.002	(0.43-23.2)	0.26	(0.26-1.30)	1.0

IHC: immunohistochemistry, LN: lymph node

OS time ranged from 2.47 to 152.1 months with median of 75.0 months. During the study period, 137 women (14.4% (137/951)) died and 814 were alive at the end of the study. HR and 95% CI for OS are shown in Table 3 and survival analyses were demonstrated in Figure 1B and 1D. Tumor size, lymph node involvement, and AJCC stage were significant prognostic factors in the analysis with all cases of breast cancers but the significance were maintained only in luminal A, TNBCs (including BLBCs and QNBC/5NPs) after subgroup analysis with molecular subtyping. Remarkably, OS in BLBCs and QNBC/5NPs, and TNBCs showed a dramatic decrease in stage III (Table 3, Additional File 1, Figure S3). Adjuvant chemotherapy was associated with prolonged OS in Luminal B (HR, 0.23; 95% CI, 0.09-0.52; P = 0.007), BLBCs (HR, 0.17; 95% CI, 0.07-0.43; P < 0.001), and TNBCs (HR, 0.41; 95% CI, 0.18-0.93; P = 0.06).

**Table 3:** Hazard ratios of overall survival for several basic characteristics by breast cancer subtyping

			Molecular subtyping											
			
	All								Five subtypes				Four subtypes	
										
Variables	(N = 951)		IHC-Luminal A (N = 486)		IHC-Luminal B (N = 123)		IHC-HER2 (N = 113)		IHC-BLBC (N = 139)		IHC-QNBC/5NP (N = 90)		IHC-TNBC (N = 229)	
		
	HR(N of event)		HR(N of event)		HR(N of event)		HR(N of event)		HR(N of event)		HR(N of event)		HR(N of event)	
	(95%CI)	p-value	(95%CI)	p-value	(95%CI)	p-value	(95%CI)	p-value	(95%CI)	p-value	(95%CI)	p-value	(95%CI)	p-value
Age Group (yr)														
< 50	1.00(85/590)		1.00(22/297)		1.00(14/87)		1.00(21/51)		1.00(17/97)		1.00(11/58)		1.00(28/155)	
≥ 50	0.99(52/361)		1.43(20/189)		1.42(8/36)		0.32(9/62)		0.92(7/42)		1.23(8/32)		1.08(15/74)	
	(0.70-1.39)	0.96	(0.78-2.61)	1.0	(0.59-3.37)	1.0	(0.15-0.71)	0.04	(0.38-2.20)	1.0	(0.49-3.04)	1.0	(0.58-2.03)	1.0
Tumor size (cm)														
≤ 2 cm	1.00(28/392)	0.005	1.00(7/228)		1.00(5/39)		1.00(6/35)		1.00(6/52)		1.00(4/38)		1.00(10/90)	
2-5 cm	2.61(87/493)		4.21(30/231)		1.29(14/75)		1.93(19/64)		1.36(12/76)		2.78(12/47)		1.86(24/123)	
	(1.70-4.00)	< 0.001	(1.94-10.1)	0.001	(0.46-3.61)	1.0	(0.77-4.83)	1.0	(0.51-3.64)	0.65	(0.89-8.63)	0.62	(0.88-3.88)	0.010
> 5cm	5.34(22/66)		6.55(5/27)		2.17(3/9)		2.46(5/14)		6.65(6/11)		7.76(3/5)		7.54(9/16)	
	(3.05-9.33)	< 0.001	(2.08-20.6)	0.008	(0.51-9.16)	1.0	(0.75-8.08)	1.0	(2.13-20.3)	0.008	(1.73-34.5)	0.046	(3.12-18.2)	< 0.001
LN involvement														
Negative	1.00(39/498)		1.00(7/246)		1.00(7/52)		1.00(12/63)		1.00(8/89)		1.00(5/48)		1.00(13/137)	
Positive	2.93(98/453)		4.88(35/240)		1.55(15/71)		2.23(18/50)		4.6(16/50)		3.96(14/42)		3.99(30/92)	
	(2.03-4.24)	< 0.001	(2.25-10.45)	< 0.001	(0.63-3.81)	1.0	(1.07-4.62)	0.26	(1.73-9.49)	0.004	(1.43-11.0)	0.064	(2.09-7.35)	< 0.001
AJCC stage														
I	1.00(10/254)		1.00(0/148)		1.00(2/19)		1.00(3/22)		1.00(2/38)		1.00(3/27)		1.00(5/65)	
II	3.29(58/467)		NA*(21/221)		1.09(10/69)		1.70(13/57)		2.74(11/78)		0.72(3/42)		1.58(14/120)	
	(1.68-6.43)	0.001			(0.23-5.01)	1.0	(0.48-5.97)	1.0	(0.61-12.3)	1.0	(0.14-3.58)	1.0	(0.57-4.40)	1.0
III	8.74(69/230)		NA*(21/117)		2.60(10/35)		3.53(14/34)		11.36(11/23)		7.85(13/21)		9.16(24/44)	
	(4.50-16.9)	< 0.001			(0.57-11.9)	1.0	(1.01-12.34)	0.37	(2.57-51.3)	0.016	(2.23-27.5)	0.005	(3.49-24.0)	< 0.001
Adjuvant chemotherapy														
Not done	1.00(35/155)		1.00(12/95)		1.00(9/20)		1.00(7/22)		1.00(6/10)		1.00(1/8)		1.00(7/18)	
Done	0.59(102/796)		0.74(30/391)		0.23(13/103)		0.75(23/91)		0.17(18/129)		2.23(18/82)		0.41(36/211)	
	(0.40-0.87)	0.008	(0.37-1.45)	1.0	(0.09-0.52)	0.007	(0.32-1.74)	1.0	(0.07-0.43)	< 0.001	(0.29-16.7)	1.0	(0.18-0.93)	0.06

* There is no event in AJCC stage I of luminal A subtype.

IHC: immunohistochemistry, LN: lymph node

### Correlation between adjusted variables and survival

We also performed multivariate analysis for DFS and OS including statistically significant variables (Table 4). Although the risk of recurrence was not significant in all cases, the HER2 and luminal B subtype was associated with recurrence. Compared to Luminal A, all other molecular subtypes were associated with a worse OS. From the survival analyses with DFS and OS, HER2 subtype was the worst prognostic subtype among four-or five subtypes (Table 4) (HR, 3.07, 95% CI, 1.86-4.87; P < 0.001). The risk of death in women with luminal B subtypes was increased 1.86-fold (95% CI, 1.11-3.11; P = 0.018), with the BLBCs the risk was increased 2.80-fold (95% CI, 1.68-4.68; P < 0.001), with the QNBC/5NPs the risk was increased 3.04-fold (95% CI, 1.75-5.26; P < 0.001), and with the TNBCs the risk was increased 2.91-fold (95% CI, 1.88-4.48; P < 0.001) compared to those with luminal A breast cancer. The likelihood ratio test between two Cox models was significant for DFS (P = 0.020) and OS (P = 0.016), indicating that adding model 1 (5-subgrouping) significantly improved the goodness of fit of the model for survival analyses in breast cancer patients. We showed that BLBC was not associated with worse prognosis than QNBC/5NP from the multivariate analyses for DFS and OS of TNBC patients (Table 5).

**Table 4:** Adjusted Hazard ratio of breast cancer survival

	Model 1 (5-subgrouping)				Model 2 (4-subgrouping)			
		
Variables	Disease-free survival		Overall survival		Disease-free survival		Overall survival	
	
	HR (95%CI)	P-value	HR (95%CI)	P-value	HR (95%CI)	P-value	HR (95%CI)	P-value
Molecular subtyping								
IHC-Luminal A					1.00		1.00	
IHC-Luminal B					1.48 (1.04-2.09)	0.027	1.86 (1.11-3.11)	0.018
IHC-HER2					1.60 (1.11-2.31)	0.011	3.07 (1.86-4.87)	< 0.001
IHC-TNBC					1.19 (0.89-1.68)	0.293	2.91 (1.88-4.48)	< 0.001
Molecular subtyping								
IHC-Luminal A	1.00		1.00					
IHC-Luminal B	1.48 (1.04-2.09)	0.028	1.86 (1.11-3.11)	0.018				
IHC-HER2	1.60 (1.11-2.31)	0.012	3.07 (1.86-4.87)	< 0.001				
IHC-BLBC	1.03 (0.68-1.56)	0.874	2.80 (1.68-4.68)	< 0.001				
IHC-QNBC/5NP	1.43 (0.93-2.18)	0.102	3.04 (1.75-5.26)	< 0.001				
Tumor size (cm)								
≤ 2 cm	1.00		1.00		1.00		1.00	
2-5 cm	1.39 (1.06-1.86)	0.02	2.26 (1.46-3.50)	< 0.001	1.39 (1.05-1.85)	0.021	2.26 (1.46-3.39)	< 0.001
> 5cm	2.19 (1.44-3.36)	< 0.001	3.21 (2.13-4.67)	< 0.001	2.16 (1.42-3.32)	< 0.001	3.19 (1.78-5.73)	< 0.001
LN involvement								
Negative	1.00		1.00		1.00		1.00	
Positive	2.40 (1.82-3.16)	< 0.001	3.17 (2.14-4.61)	< 0.001	2.41 (1.83-3.18)	< 0.001	3.17 (2.14-4.61)	< 0.001
Adjuvant Chemotherapy								
Not done	1.00		1.00		1.00		1.00	
Done	0.70 (0.50-0.99)	0.049	0.34(0.22-0.50)	< 0.001	0.70 (0.50-0.99)	0.048	0.34 (0.22-0.50)	< 0.001

IHC: immunohistochemistry

**Table 5:** Adjusted Hazard ratio of breast cancer survival in IHC-TNBC

	Disease-free survival		Overall survival	
	
	HR (95%CI)	P-value	HR (95%CI)	P-value
Molecular subtyping				
IHC-BLBC	1.00		1.00	
IHC-QNBC/5NP	1.43 (0.84-2.45)	0.188	1.20 (0.65-2.22)	0.558
Tumor size (cm)				
≤ 2 cm	1.00		1.00	
2-5 cm	1.44 (0.76-2.73)	0.258	1.61 (0.75-3.46)	0.219
> 5cm	3.06 (1.27-7.35)	0.012	4.07 (1.56-10.5)	0.004
LN involvement				
Negative	1.00		1.00	
Positive	2.54 (1.44-4.48)	0.001	3.83 (1.92-7.65)	< 0.001
Adjuvant Chemotherapy				
Not done	1.00		1.00	
Done	0.54 (0.23-1.28)	0.161	0.31(0.13-0.76)	0.011

### Chemotherapy effects on the subtypes

**Figure 2:**
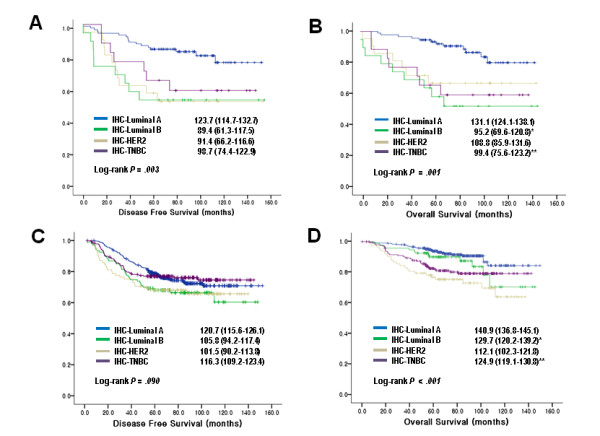
**Association between chemotherapy and four-subtypes of breast cancers**. Kaplan-Meier curves of disease-free survival (A and C) and overall survival (B and D). Cases without chemotherapy (A and B) and with chemotherapy (C and D) (Estimated mean survival with 95% CI). *P < 0.001, **P = 0.027

**Figure 3:**
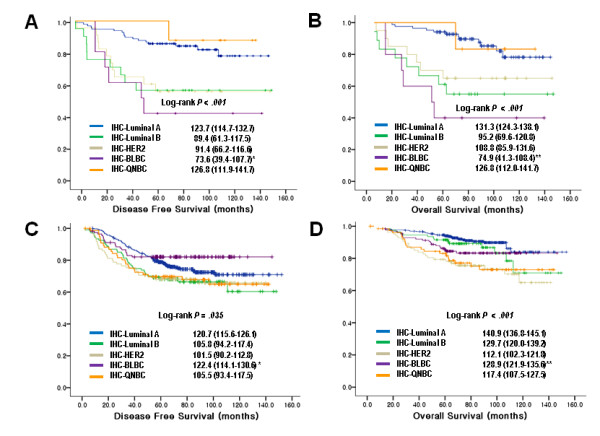
**Association between chemotherapy and five-subtypes of breast cancers**. Kaplan-Meier curves of disease-free survival (A and C) and overall survival (B and D). Cases without chemotherapy (A and B) and with chemotherapy (C and D) (Estimated mean survival with 95% CI). *P = 0.001, **P < 0.001

When the patients were divided into two groups depending on the adjuvant chemotherapy, the survival analysis revealed that patients with luminal B and TNBCs had overall survival benefit from chemotherapy (P < 0.001 for luminal B, P = 0.027 for TNBCs) (Figure 2). From the five-subtype analysis, the BLBCs without adjuvant chemotherapy had a shortest DFS and OS showing dramatic survival gain after chemotherapy (P = 0.001 for DFS, P < 0.001 for OS) (Figure 3). On the other hand, QNBC/5NP did not show chemotherapy benefit in both DFS and OS. However, this was difficult to interpret due to small numbers etc (only 1 event in the QNBC/5NP group) (Figure 3). Furthermore, multivariate analysis of BLBC patients for DFS and OS including tumor size and lymph node involvement confirmed survival gain of adjuvant chemotherapy (Table 6). The survival gain with anthracycline-based and non-anthracycline-based adjuvant chemotherapy did not show statistical difference in BLBCs (data not shown).

**Table 6:** Adjusted Hazard ratio of breast cancer survival in IHC-BLBC

	Disease-free survival		Overall survival	
	
	HR (95%CI)	P-value	HR (95%CI)	P-value
Tumor size (cm)				
≤ 2 cm	1.00		1.00	
2-5 cm	1.49 (0.51-2.61)	0.740	1.48 (0.57-3.87)	0.422
> 5 cm	1.72 (0.55-5.32)	0.349	2.45 (0.73-8.24)	0.147
LN involvement				
Negative	1.00		1.00	
Positive	3.21 (1.49-6.89)	0.003	4.78 (1.95-11.73)	0.001
Adjuvant Chemotherapy				
Not done	1.00		1.00	
Done	0.34 (0.12-0.88)	0.028	0.22(0.08-0.59)	0.011

### Molecular subtyping in public microarray datasets

**Figure 4:**
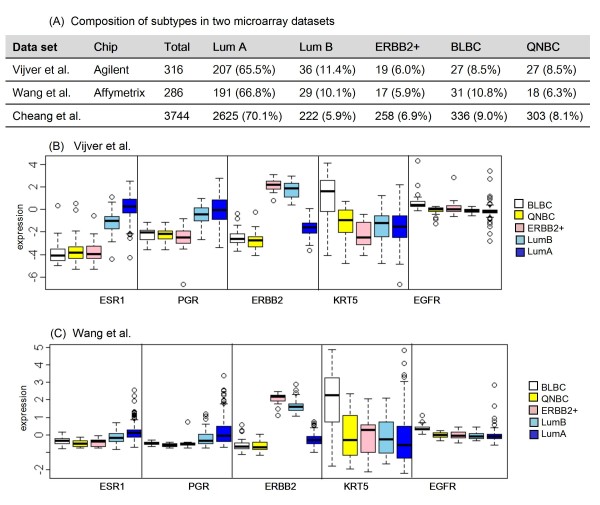
**Comparison of the expression levels of five genes according to five subtypes in the microarray data sets**. (A) Proportion of five breast cancer subtypes in two large-sized microarray data sets. (B) Expression levels of the five markers according to the subtypes in Vijver et al. (C) Expression levels of the five markers according to the subtypes in Wang et al. Distribution of expression levels of each marker was described with box plots. The bottom and top of the box are the 25^th ^and 75^th ^percentile, and the band near the middle of the box is the 50th percentile.

In order to support our data that TNBC is substantially different from BLBC, we explored the mRNA expression levels of the five genes (ESR1, PGR, ERBB2, KRT5 and EGFR) according to five subtypes in two large-sized microarray datasets. We classified each dataset into five subtypes from the definition of subtypes described in Methods. From the classification, the proportion of five subtypes in the two microarray datasets turned out to be very similar, and it was similar to the proportion of subtypes in Cheang et al.'s data set as well (Fig 4A). The proportion of QNBC/5NP within TNBC was about 36% and 50% in each microarray dataset, and our data set showed similar proportion which was about 40%. The expression patterns of each gene in five subtypes were consistent across the datasets displaying the clear characteristics of each subtype. Elevated expression of KRT5 and EGFR was a distinct feature of BLBC discriminating it from the other subtypes, and their high expression in BLBC were independent from the expression of other three genes - ESR1, PGR and ERBB2 - which showed no difference between BLBC and QNBC/5NP (Figure 4B and 4C). KRT5 showed a wide range of expression level across samples, while the expression range of EGFR was relatively very narrow in both datasets, and most of the TNBC samples showing high expression of KRT5 accompanied high expression of EGFR (data not shown). From the overall expression patterns of five genes, KRT5 and EGFR seemed to be one of the major causes of the heterogeneity of TNBC, and a large portion of TNBC was non-BLBC subtypes supporting that TNBC should not be replaced interchangeably with BLBC.

## Discussion

In summary, with 951 breast cancer cases, we observed that TNBCs showed a poor OS prognosis, showing higher nuclear and/or histological grade, next to the HER2 subtypes. The worse OS was observed in QNBC/5NPs among TNBCs. In previous studies, the frequency of TNBCs and BLBCs has ranged from 17.1% to 30.5% and 8.0% to 55.7% respectively, depending upon the definition or criteria used [21]. The difference of the frequency might be explained with the method and criteria of the IHC interpretation. In this study, the proportion of BLBCs was 14.6% (60.7% of TNBCs), which was similar with that (14.7%) in the previous Kim et al.'s study, which used four basal makers (CK5, CK14, EGFR, and c-kit) [15]. We used two basal markers (CK5/6, EGFR) in accordance with the Carolina Breast Cancer Study [17].

When the TNBCs tumors were divided into BLBCs and QNBC/5NPs based upon basal markers, QNBC/5NP had the poorer OS in our study. The Luminal A and BLBCs had the best DFS. Previous studies have shown that the expression of basal markers (basal cytokeratins and EGFR) in TNBCs also correlates with a worse prognosis and identifies a clinically distinct subgroup within the TNBCs [9,10,13]. In our study, CK5/6 was a poor prognostic marker but EGFR was not (data not shown). In Cheang et al.'s data from 3,744 cases, 17% were TNBCs and 9% were BLBCs, using the five-marker method [13]. They showed that the poor prognosis of the TNBCs is conferred almost entirely by those tumors positive for basal markers, that is, BLBCs. In our study, the clinicopathologic characteristics of TNBCs were maintained in BLBCs after dividing into two groups (BLBCs and QNBC/5NPs), but the poor prognosis of the TNBCs was primarily due to the QNBC/5NPs.

Without chemotherapy, BLBCs were worst prognostic subtype in both DFS and OS. Interestingly, BLBCs showed dramatic clinical benefit from both anthracycline-based and CMF adjuvant chemotherapy. On the other hand, adjuvant chemotherapy showed little clinical benefit for QNBC/5NP in this study. However, this result simply reflects that some QNBC/5NP patients may not benefit from standard chemotherapy. Several studies of breast cancer patients treated with neoadjuvant anthracycline-based chemotherapy have reported higher response rates in TNBCs compared to luminal breast cancers [22,23]. In an adjuvant setting, the HR for relapse or death among patients with BLBCs (HR, 0.54; 95% CI, 0.27-1.08) was reduced after treatment with anthracycline-based chemotherapy [24]. Along with those results, our study implies that the basal markers may be useful to identify TNBC patients who are most likely to benefit from anthracycline-containing adjuvant chemotherapy.

The finding that QNBC/5NPs do worse with regard to DFS and OS than BLBCs is different from the findings by Cheang et al. [13], Carey et al. [17] and Rakha et al. [8]. This difference might be due to the different proportion of breast cancer patients with no chemotherapy (Cheang et al: BLBCs treated/untreated: 103/179, here: 129/10). Furthermore, our results could be explained by the fact that BLBCs have been reported to benefit from chemotherapy [24]. Similarly the finding that BLBCs do just as well on CMF as on anthracycline-based chemotherapy, while QNBC/5NPs do worse on chemotherapy contradicts with the findings of Cheang et al. This discrepancy could be drawn from the different cohort, especially Asian population in this study, or small patient numbers used in this study.

Since 2001, breast cancer has been the most common cancer in women in Korea [25]. Recently, Rhee et al. reported that, in node-negative breast cancers, TNBC has a higher relapse rate and more aggressive clinicopathologic characteristics than non-TNBC [26]. However, the author did not divide the cases into five subtypes including BLBCs. Kim et al. reported the clinicopathologic significance of the BLBCs based on the expression of basal cytokeratins [15] indicating that BLBCs were associated with high histological and/or nuclear grades, but no statistically significant survival differences were evident between BLBCs and those of other subtypes. BLBCs and/or TNBCs have been known to be associated with BRCA mutations, which have been investigated mostly in western countries. In the contrary, in Korea, the mutation rates of BRCA1 and 2 are extremely low, which implies that most BLBCs or TNBCs are not associated with BRCA mutations. In the population-based Carolina Breast Cancer Study, the prevalence of the BLBCs and luminal A subtypes was strongly influenced by race and menopausal status [17]. Some different tumorigenic mechanism may be involved in BLBCs or TNBCs breast cancers in Asia.

This study was limited in that the cohort is derived from a single institution in a large city (Seoul, Korea). Considering protein profiling by IHC is not homogenous in whole sections and the results of IHC from the TMAs usually give the lower frequencies than from the whole sections, BLBCs by TMA-based IHC can be contaminated with luminal A subtypes of relatively high proportion and their prognosis can be a little bit better than using whole sections. To rule out this possibility, ER and PR status were determined by assessment of whole sections of the tumor, not from cores as in TMA, because the patient was treated with anti-hormonal therapy based upon the result from whole sections. The remaining IHC results were from the TMA setting with two copies of 2-mm core to represent above at least 98% of the whole lesion (theoretical area is 8π), since usual one 1-mm core (theoretical area is π) represents 91% of the whole lesion and three cores (theoretical area is 3π) do 98% [27]. In this study, positive rate of ER and/or PR might be a little bit higher than the studies that are evaluated in TMA, implying that luminal A or B subtypes could be more prevalent in this study.

The QNBC/5NPs was observed in about 6~8% of total samples from publicly available breast cancer microarray datasets, and about 40% of TNBCs were non-BLBCs, which supports that TNBCs is not synonymous with BLBCs. TNBCs have the poorer associated prognosis of breast cancer subtypes, a finding which is consistent with earlier studies. By adding EGFR and CK5/6 as basal markers, QNBC/5NPs were found to have the worse outcomes among TNBCs. Kreike et al. showed that, based on the gene-expression profiling, BL-subtype tumors (classified as TN tumors in their study) are heterogeneous and can be subdivided into at least five distinct subtypes [7]. Bertucci et al. called for caution in the interpretation of ongoing trials and the selection of patients for future trials based on their data, which showed that TNBCs represent a more heterogeneous group than BLBCs since TNBCs include basal and non-basal tumors, which are very different at the histological and molecular levels, most notably in regard to mRNA expression of molecules targeted by specific therapies that are under evaluation in clinical trials [28]. The risk of regarding the two tumor types as the same is that TNBCs included in clinical trials may not be identical to BLBCs, possibly leading to a falsely negative conclusion of unresponsiveness to drugs that may actually be capable of treating true BLBCs [28]. Bertucci et al. also revealed that BLBCs are a more homogenous group than TNBCs. The incomplete concordance between BLBCs and TNBCs has been reported by using various immunohistochemical definitions, including the ER-, HER2-, EGFR+, and/or CK5/6+ immunohistochemical profile, which is currently considered to be the most reliable definition [2]. In the majority of previous studies, the basal status of many TNBCs was thought to confer a poorer clinical outcome when compared to non-basal TNBCs; however, our results suggest that the QNBC/5NPs may be primarily responsible for conferring poor clinical outcomes.

## Conclusions

In conclusion, TNBCs and BLBCs were associated with high histologic and/or nuclear grades. However, in contrast to previous data, QNBC/5NPs showed worse prognosis in this study, which might be from the poor response to the adjuvant chemotherapy. Based on these findings, the prognostication and the identification of subgroup of patients for therapeutic classification should be re-considered with more applicable markers. Furthermore, our study suggests that patients with QNBC/5NP might not benefit from anthracycline-based chemotherapy.

## Competing interests

The authors declare that they have no competing interests

## Authors' contributions

YLC and EO: designed the study, carried out the experiments, interpreted the data and wrote the manuscript; SP, YHP, EYC and YHI: critically revised the manuscript; JSC, YK and YCH: analyzed the immunohistochemical data and drafting of the manuscript; KS: carried out the experiments and data acquisition; JEL, JHK, SJN, JHY: participated in design and data collection of the data; YKS: participated in study design and coordination, data analysis, data interpretation and drafting of the manuscript. All authors read and approved the final manuscript.

## Pre-publication history

The pre-publication history for this paper can be accessed here:

http://www.biomedcentral.com/1471-2407/10/507/prepub
