# Correction: triple-negative, basal-like, and quintuple-negative breast cancers: better prediction model for survival

**DOI:** 10.1186/1471-2407-11-13

**Published:** 2011-01-12

**Authors:** Yoon-La Choi, Ensel Oh, Sarah Park, Yeonju Kim, Yeon-Hee Park, Kyug Song, Eun Yoon Cho, Yun-Chul Hong, Jong Sun Choi, Jeong Eon Lee, Jung Han Kim, Seok Jin Nam, Young-Hyuck Lim, Jung-Hyun Yang, Young Kee Shin

**Affiliations:** 1 Laboratory of Cancer Genomics and Molecular Pathology; 2 Department of Pathology, Samsung Medical Center, Sungkyunkwan University School of Medicine, Seoul, Korea; 3 Research Institute of Pharmaceutical Science; 4 Interdiciplinary Program of Bioinformatics, Department of Pharmacy, Seoul National University College of Pharmacy, Seoul, Korea; 5 Division of Medical Oncology, Department of Internal Medicine, Seoul St. Mary's hospital, Catholic University, Seoul, Korea; 6 Cancer Early Detection Branch, National Cancer Control Research Institute, National Cancer Center, Goyang, Korea; 7 Department of Internal Medicine, Samsung Medical Center, Sungkyunkwan University School of Medicine, Seoul, Korea; 8 Department of Preventive Medicine, Seoul National University College of Medicine, Seoul, Korea; 9 Department of Pathology, Dongguk University Ilsan Hospital, Dongguk University College of Medicine, Goyang, Korea; 10 Department of Surgery, Samsung Medical Center, Sungkyunkwan University School of Medicine, Seoul, Korea

## Correction

After the publication of this work [1], we found that there were some mistakes in calculating the percentage of composition in Table 1 (1). Clinicopathologic characteristics of breast cancer subtypes. We are therefore providing the revised Table 1, with the updated data for rows 'Mucinous carcinoma', 'Metaplastic carcinoma' and 'Others'. In the sub-content of Table 1, 'Histological type', the total number of 'Others' was corrected from 18 to 16, and the composition of 'Others' type was slightly changed according to breast cancer subtypes. For IHC-Her2 subtype, the number of 'Others' was changed from 4 to 3, and 6 cases which were previously unidentified were assigned to corresponding subtypes. One case to IHC-BLBC, 2 cases to IHC-QNBC/5NP and 3 cases to IHC-TNCB. There was no effect on statistical analysis with the correction.

**Figure 1:**
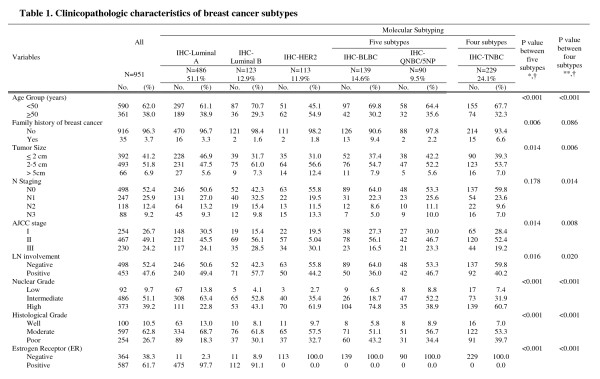
revised Table 1

## Pre-publication history

The pre-publication history for this paper can be accessed here:

http://www.biomedcentral.com/1471-2407/11/13/prepub
